# Reading-Induced Shifts in Speech Perception in Dyslexic and Typically Reading Children

**DOI:** 10.3389/fpsyg.2019.00221

**Published:** 2019-02-07

**Authors:** Linda Romanovska, Roef Janssen, Milene Bonte

**Affiliations:** Maastricht Brain Imaging Center, Department Cognitive Neuroscience, Faculty of Psychology and Neuroscience, Maastricht University, Maastricht, Netherlands

**Keywords:** reading development, dyslexia, letter-speech sound coupling, recalibration, adaptation

## Abstract

One of the proposed mechanisms underlying reading difficulties observed in developmental dyslexia is impaired mapping of visual to auditory speech representations. We investigate these mappings in 20 typically reading and 20 children with dyslexia aged 8–10 years using text-based recalibration. In this paradigm, the pairing of visual text and ambiguous speech sounds shifts (recalibrates) the participant’s perception of the ambiguous speech in subsequent auditory-only post-test trials. Recent research in adults demonstrated this text-induced perceptual shift in typical, but not in dyslexic readers. Our current results instead show significant text-induced recalibration in both typically reading children and children with dyslexia. The strength of this effect was significantly linked to the strength of perceptual adaptation effects in children with dyslexia but not typically reading children. Furthermore, additional analyses in a sample of typically reading children of various reading levels revealed a significant link between recalibration and phoneme categorization. Taken together, our study highlights the importance of considering dynamic developmental changes in reading, letter-speech sound coupling and speech perception when investigating group differences between typical and dyslexic readers.

## Introduction

Reading is a complex cognitive skill most of us learn within the first decade of life. While there is some variability in how smoothly this learning process goes, most children learn to correctly associate corresponding letters and speech-sounds after 1 year of reading instruction (Blomert, 2011) and continue refining the newly acquired skill over a protracted period throughout primary school (Maurer et al., 2006; Brem et al., 2009; Froyen et al., 2009; Ben-Shachar et al., 2011). However, 5–10% of children show particular difficulties in learning to read and are diagnosed with developmental dyslexia, a learning difficulty characterized by impaired reading fluency and spelling despite adequate intelligence, motivation and schooling (Lyon et al., 2003).

A number of theories have been proposed to describe the underlying mechanisms of developmental dyslexia, ranging from phonological (Snowling, 1980; Shaywitz et al., 1998; Lyon et al., 2003), to audio-visual (Blomert, 2011; Kronschnabel et al., 2014; Aravena, 2017), visual (Bosse et al., 2007; Vidyasagar and Pammer, 2010), auditory (Tallal, 2004; Vandermosten et al., 2010), magnocellular (Ramus, 2003) and cerebellar (Fawcett and Nicolson, 1999) deficits. However, most theories converge in acknowledging that dyslexic readers typically exhibit difficulties in phonological processing and that the formation of robust letter-speech sound mappings is essential to fluent reading acquisition. Here we explore letter-speech sound mappings in typically reading children and children with dyslexia using a newly developed short-term audio-visual learning paradigm called text-based recalibration.

Support for a deficit in letter-speech sound integration in dyslexic readers largely comes from studies comparing the processing of congruent versus incongruent letter-speech sound stimuli. Indeed, both behavioral (Snowling, 1980; Blomert and Willems, 2010; Aravena et al., 2013) and brain activity studies (Blau et al., 2009, 2010; Froyen et al., 2011; Žarić et al., 2014, 2015; Jones et al., 2016; Moll et al., 2016) have shown that children and adults with dyslexia process letter speech sound pairs differently from typical readers (but see Nash et al., 2016; Clayton and Hulme, 2017). In a series of EEG studies in the relatively transparent Dutch orthography, these differences were observed in audio-visual mismatch negativity (MMN) and late negativity (LN) responses at a 100–200 ms and 600–750 ms latency following an audio-visual deviant stimulus in a sequence of standards (Froyen et al., 2009, 2011; Žarić et al., 2014). The audio-visual MMN and LN responses can be seen as an indirect measure of letter-speech sound integration, for only if the auditory and visual modalities have been properly processed and integrated, they will yield a mismatch response. Studies by Froyen and Žarič and colleagues have revealed that children with dyslexia show a reduced audiovisual MMN and/or LN response compared to typically reading children, pointing to a reduced integration of letters and speech sounds. Furthermore, the latency of these responses has been found to scale with reading fluency and remediation, respectively (Žarić et al., 2014, 2015). Concordantly, in functional magnetic resonance imaging (fMRI) studies, superior temporal cortical (STC) activity of children (Blau et al., 2010) and adults (Blau et al., 2009) with dyslexia, as well as pre-readers at familial risk of dyslexia (Karipidis et al., 2017), has been found to show less sensitivity to letter-speech sound (in)congruency compared to typical readers. Taken together these findings indicate deviant letter-speech sound processing and integration processes in dyslexic readers.

However, the manner in which stimulus (in)congruency is processed may be influenced by a number of factors, including individual differences in the level of reading skills (Plewko et al., 2018), or phoneme perception (Basu Mallick et al., 2015), but also more general factors such as attentional focus (Talsma and Woldorff, 2005), task characteristics (Basu Mallick et al., 2015) or familial risk for dyslexia (Maurer et al., 2003). A complementary approach to investigate letter-speech sound coupling can be found in (phonetic) recalibration paradigms, in which the perceived identity of an ambiguous speech sound is biased in the direction of previously presented disambiguating context information. This context information can consist of lip-read speech (Bertelson et al., 2003; Vroomen and Baart, 2012), lexical (spoken word) context (Norris et al., 2003), overt or imagined speech articulation (Scott, 2016), or, most relevant for our current study, visual text (Bonte et al., 2017; Keetels et al., 2018). In the classical recalibration paradigm an ambiguous speech sound /a?a/ midway between /aba/ and /ada/ is combined with a disambiguating video of a speaker articulating ‘aba’ or ‘ada’ to bias the perception of the ambiguous sound toward the video. Thus, repeated presentation of a speaker articulating ‘aba’ while playing the /a?a/ sound, shifts participants’ subsequent perception of this ambiguous sound toward /aba/. Similarly, a speaker articulating ‘ada’ shifts later perception toward /ada/. Recalibration thus involves an ‘attracting’ perceptual bias where participants perceive phoneme boundary shifts toward the visual information. The induced bias (recalibration) is typically described as a multi-sensory perceptual effect that has been found to be minimally influenced by higher-level task demands (Baart and Vroomen, 2010). In contrast, an opposite ‘repulsive’ perceptual bias (or auditory selective adaptation) is induced after repeated presentation of the same videos together with clear speech sounds. That is, after exposure to a speaker articulating ‘aba’ together with clear /aba/ speech sounds, the ambiguous /a?a/ sound is more likely to be perceived as /ada/ (and ‘ada’ articulation more often leads to /aba/ perception; Bertelson et al., 2003; Vroomen et al., 2004; Keetels et al., 2016). Phonetic recalibration with lip-read speech has been reliably shown in typically reading adults (Bertelson et al., 2003) and 8-year-old children but not in 5-year-old children, suggesting a developmental build-up of the effect (van Linden and Vroomen, 2008). A similar but delayed developmental trend has been reported in the adaptation effect, with robust effects observed in adults (Bertelson et al., 2003; Vroomen et al., 2004, 2007; Baart and Vroomen, 2010) but not in 5–10 year-old children (Sussman and Carney, 1989; Sussman, 1993).

To investigate potential differences in letter speech-sound mappings between children with dyslexia and typically reading children, we use a recent modification of the recalibration paradigm which employs visual ‘aba’ or ‘ada’ text to bias the perception of ambiguous /a?a/ speech sounds (Keetels et al., 2016, 2018; Bonte et al., 2017). Most interestingly, while both videos and text were recently shown to elicit significant recalibration effects in typically reading adults (Keetels et al., 2018), adults with dyslexia only showed significant recalibration with videos, but not with text (Keetels et al., 2018), suggesting a specific deficit in the audiovisual mapping of letters and speech sounds. Here, we use text-based recalibration to investigate letter-speech sound mapping in 8–10 year-old typically reading children and children with dyslexia. While the nature of the study was exploratory, as text-based recalibration has not been previously studied in children, we expected to replicate the findings of Keetels et al. (2018) and to observe significant recalibration effects only in typical readers. We also explored potential links between recalibration effects and individual differences in reading proficiency (accuracy and fluency) and in categorical speech perception (phoneme categorization slope). In addition, we employ an adaptation task with clear /aba/ and /ada/ stimuli providing both a baseline with respect to potential response strategies and a test for potential developmental changes in speech adaptation (van Linden and Vroomen, 2008).

## Materials and Methods

### Participants

Twenty children with dyslexia (mean age 8.5 ± 0.82 years; 9 females) were recruited from a specialized institute for dyslexia and reading problems, and fifty-six typically reading children (mean age 8.4 ± 0.94 years; 34 females) from local elementary schools. Parents gave written informed consent for participation in the study. To perform group comparisons and run statistical analyses, a subset of twenty typically reading children were matched for age, gender and scores on a non-verbal subtest (block design) of the Dutch version of the Wechsler Intelligence Scale for Children-III (WISC-III-NL; Kort et al., 2005) to the children with dyslexia, group characteristics and comparisons using one-way ANOVA are shown in Table 1. All children were native Dutch speakers with no reported hearing impairments, normal or corrected to normal vision, and no history of diagnosed neurological disorders. The dyslexia diagnosis was given by the institute based on the results of extensive cognitive psycho-diagnostic testing and results of standardized reading measures. Children received a small present as participation reward. The experiment was approved by the ethics committee of the Faculty of Psychology and Neuroscience, Maastricht University.

**Table 1:** Descriptive statistics of typical and dyslexic readers.

N Age Gender ratio(m/f)	Dyslexic readers 20 8.60 (0.94) 11:9			Typical readers 20 8.70 (1.13) 11:9			Dyslexic vs. Typical readers *t*(1,38) = -0.30, *p* = 0.76	
**Word reading - accuracy [%]**	***M***	***SD***	**Range**	***M***	***SD***	**Range**	***F*(1,39)**	***p***
3DM High frequency words	94.69	7.13	73–100	99.79	0.65	97–100	10.16	0.003
3DM Low frequency words	89.48	10.26	63–100	99.07	3.21	85–100	15.90	0.000
3DM Pseudo words	83.88	13.03	50–100	91.82	6.17	81–100	6.07	0.018
3DM Total	90.28	8.35	63–98	97.72	2.11	91–100	14.92	0.000
3DM Total words [T]	38.75	11.32	20–57	55.20	5.87	40–63	33.24	0.000
**Word reading - fluency [T]**								
3DM High frequency words	33.45	7.97	21–49	58.85	11.48	36–80	66.02	0.000
3DM Low frequency words	33.55	5.70	23–45	57.50	11.26	32–78	71.94	0.000
3DM Pseudo words	35.25	6.07	24–46	55.30	12.36	34–79	42.36	0.000
3DM Total words	33.10	6.15	22–45	57.95	11.96	33–80	68.20	0.000
**IQ norm scores**								
Verbal (similarities)	11.20	2.26	7–15	13.95	2.89	9–18	11.22	0.002
Non-verbal (block design)	11.15	3.45	5–18	12.50	3.57	7–19	1.47	0.232

### Literacy Skills

Each participant performed a computerized reading task of the 3DM (Dyslexia Differential Diagnosis; Blomert and Vaessen, 2009). The task comprised three subtasks including reading of high frequency words, low frequency words and pseudo words. Instructions of the reading task were simultaneously presented on the computer screen and aurally through over-ear headphones. The participant was asked to read the (pseudo)words as quickly and accurately as possible. For each subtask the participant had a time limit of 30 s to read. Reading accuracy was determined by calculating the proportion of correctly versus incorrectly read words within the given time limit. Reading fluency was calculated as the number of correctly read words within the given time limit for the whole task as well as per subtask.

### Experimental Design and Procedure

#### Stimuli

The speech stimuli consisted of recordings of a native male Dutch speaker pronouncing the speech sounds /aba/ and /ada/ (see Bertelson et al., 2003 for a detailed description). Both speech sounds lasted 650 ms and were used to create a nine-token continuum (BD1-BD9) ranging from a clear /aba/ sound to a clear /ada/ sound by changing the second formant (F2) in eight steps of 39 Mel using PRAAT software (Boersma and Weenink, 2001). The visual stimuli consisted of the written counter-parts of the speech sounds, namely ‘aba’ and ‘ada’ text presented in white at the center of a black screen in ‘Times New Roman’ font (font size 50). The auditory and visual stimuli were presented using Presentation software (Version 17.2, Neurobehavioral Systems, Inc., Berkeley, CA, United States).

All children completed the pre-test, recalibration and adaptation tasks. The children with dyslexia completed these tasks in a quiet room at the specialized dyslexia institute, whereas the typically reading children were tested in a quiet room at their school. All tasks were performed on a laptop computer with the auditory stimuli presented at a comfortable listening level over noise-canceling headphones (SONY MDR-7509HD).

#### Pre-test

**FIGURE 1:**
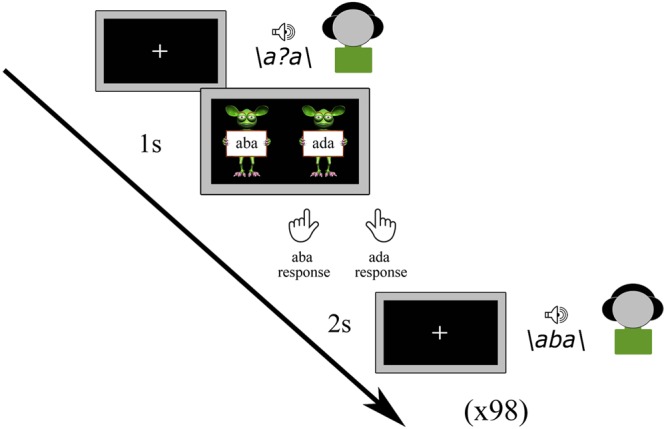
Pre-test.

Prior to the main experimental tasks, all participants completed a pre-test in which all nine tokens of the /aba/ - /ada/ continuum were presented a total of 98 times in a randomized order. The children were instructed to listen to each sound carefully and to indicate which sound they heard by pressing the left (/aba/) or right (/ada/) shift button with the left or right index finger, respectively, following a response cue (Figure 1). The response cue consisted of ‘aba’ (left) and ‘ada’ (right) text held up by cartoon monsters created using the Monster Workshop content pack of the iClone 6 software^1^. No emphasis was put on speed, and it was furthermore emphasized that there were no correct or incorrect responses. While the speech sounds were played, children viewed a black screen with a white fixation cross, which was followed by the response screen (cartoon monsters) after 1 s and terminated when children provided a response. The subsequent speech sound was presented 2 s after a response was given. The total duration of the pre-test was approximately 5 min.

The results of the pre-test were used to determine the most ambiguous speech sound for each participant. This was done based on the proportion of /aba/ responses to each token along the /aba/-/ada/ continuum and was identified as the sound with a response proportion of /aba/ versus /ada/ closest to 0.5. This individually determined most ambiguous sound was subsequently used in the audiovisual exposure blocks of the recalibration task as well as in the post-test trials of the recalibration and adaptation tasks. In the post-trials, next to the most ambiguous sound, we also presented its flanking sounds /a?a/+1 and /a?a/-1 on the /aba/-/ada/ continuum.

The pre-test served two purposes: (1) to determine the most ambiguous sound for each participant, and (2) to allow for the investigation of the phoneme categorization slope in each group. Previous research has indicated that adult readers with dyslexia perceive speech sounds less categorically compared to typical readers (Ahissar, 2007; Baart et al., 2012). Thus, the results of the pre-test allow us to investigate whether these findings extend to our sample of children with dyslexia and typically reading children.

#### Recalibration Task

**FIGURE 2:**
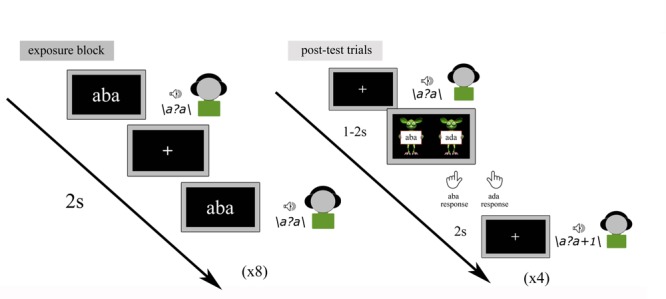
Text-based recalibration paradigm.

The text-based recalibration paradigm is composed of audio-visual exposure blocks and subsequent auditory-only post-test trials (Figure 2). During each audio-visual exposure block, the children were presented with 8 repetitions of either the text ‘aba’ or ‘ada’, paired with the individually determined ambiguous speech sound /a?a/. The speech sound and visual text were presented simultaneously (relative SOA of 0 ms) and auditory stimuli had a duration of 650 ms, while text was presented for 1 s. The inter-trial interval between subsequent audio-visual exposure trials was set to 2 s. During the audio-visual exposure blocks, children were instructed to pay close attention to the speech sounds and text without providing a response.

Each exposure block was followed by four auditory-only post-test trials. The four post-test sounds were presented in a randomized order with the individually determined most ambiguous /a?a/ sound presented twice and each of its flanking sounds /a?a/+1 and /a?a/-1 on the /aba/-/ada/ continuum, presented once. Each post-test sound was followed by a response cue consisting of ‘aba’ and ‘ada’ texts held by cartoon monsters (Figure 2).

Children were instructed to listen to each sound carefully and to make forced-choice /aba/-/ada/ judgments by pressing the left/right shift button with the left/right index finger, respectively, once the cartoon monsters appeared. Identical to the pre-test, no emphasis was put on speed and it was further emphasized that there were no incorrect responses. All responses were self-paced. The onset of the response picture was jittered 1–2 s in relation to the post-test sound and was terminated upon the button-press. Post-test trials were presented with an inter-trial interval of 2 s after the participant had provided a response.

The recalibration task was divided into 2 6-min runs, both consisting of 10 ‘aba’ and 10 ‘ada’ exposure blocks, each followed by 4 post-test trials amounting to 40 post-test trials for each type of exposure block.

#### Adaptation Task

The adaptation task was identical to the recalibration task in all aspects except for the speech sounds used in the exposure blocks. Here, the clear /aba/ and /ada/ sounds were combined with the corresponding ‘aba’ and ‘ada’ text, creating congruent audio-visual stimuli in the exposure blocks. The task instructions and stimulus timings were all identical to those of the recalibration task. The adaptation task allowed us to explore auditory adaptation and served as a control for potential response strategies that children may employ.

All children performed the pre-test followed by 2 runs of the recalibration task and 2 runs of the adaptation task. The task order was kept constant across all participants. The reason for this fixed order instead of counterbalancing was threefold: (1) because we were interested in audiovisual learning, the recalibration blocks were of primary interest, with the adaptation blocks serving as a control, (2) this behavioral experiment served as a preparation of a longitudinal fMRI project where we only included text-based recalibration, and (3) initial pilot results suggested interference from adaptation blocks to subsequent recalibration blocks but not vice versa. This finding is in line with the observation of short-lived audio-visual recalibration effects compared to longer lasting adaptation effects (Vroomen and Baart, 2012).

### Statistical Analysis

The data were assessed for statistical significance using repeated measures ANOVA (SPSS version 24.0, IBM Corp., Armonk, NY, United States). The ANOVA model included the type of task (recalibration vs. adaptation), type of exposure (‘aba’ text vs. ‘ada’ text), post-test sounds (/a?a/, /a?a/ +1, /a?a/-1) as within subjects factors and group (dyslexic vs. typically reading) as between subjects factor. The differences in average /aba/ versus /ada/ response proportions (aftereffects) following the two types of exposure blocks were further assessed using paired-samples *t*-tests. For the conditions in which the sphericity assumption was violated, the degrees of freedom were adjusted using the Greenhouse-Geisser correction.

The fit of the pre-test slopes was estimated using the Slope Fitting Tool in MATLAB 2016a (The MathWorks, Inc., Natick, MA, United States). Based on previous literature, a custom logistic function (Function 1) was used to obtain partial R^2^ values and evaluate the goodness of fit of individual as well as group-level categorization slopes (McMurray and Spivey, 2000; Ley et al., 2012). Subsequently, the non-linear least squares solver in MATLAB was employed to obtain the slope value (c in Function 1) that provided the best fit to the data and yielded the smallest sum of squares. To optimize the outcome, the results of the fitting procedure were restricted for each of the variables in Function 1 to 0 ≤ a ≥ 10, -10 ≤ b ≥ 10, -10 ≤ c ≥ 10, -9 ≤*d* ≥ 18. The best fit was determined by running 30 iterations of the slope fitting procedure and taking the slope value with the smallest sum of squares. The number of iterations was verified by replicating the procedure multiple times.

(1) $$\begin{array}{c} y=\frac{a}{{1+e}^{\frac{-(x-d)}{c}}}+b \end{array}$$

Function 1: a, amplitude of the function; b, lowest asymptote of y-axis; c, slope of the function; d, location of the category boundary.

To investigate a potential link between recalibration/ adaptation aftereffects, pre-test slope and behavioral reading measures, linear regression analyses were performed in R 3.4.1 (R Development Core Team, 2013). In addition, all statistical analyses were also performed on the complete sample of controls to assess the reliability of our findings within a larger sample of typical readers of various reading levels.

## Results

### Pre-test

**FIGURE 3:**
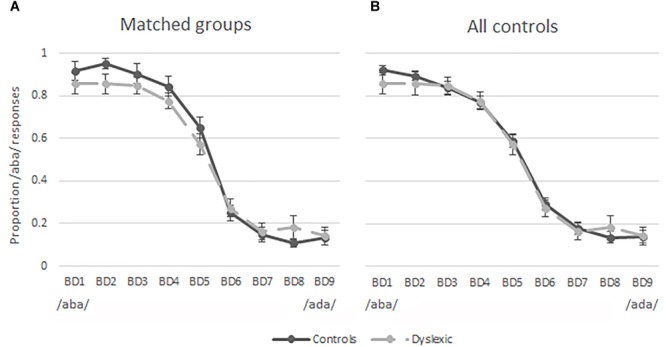
Pre-test results plotted as proportion of /aba/ responses for each token along the continuum. Solid lines = typical readers, dashed lines = dyslexic readers, bars = standard errors; **(A)** matched groups; **(B)** 20 dyslexic readers and 56 typical readers.

The results of the pre-test were used to investigate the categorical perception of the nine auditory tokens employed in this study. Figure 3A shows the proportion of /aba/ responses per sound stimulus in children with dyslexia (dashed line) and the matched typically reading control children (solid line). These figures indicate similar categorical perception of speech sounds in the groups of typically reading children and children with dyslexia. This observation was confirmed by a 9 auditory token × 2 (Group) repeated measures ANOVA. The ANOVA revealed an expected main effect of sound [*F*(2,100) = 135.03, *p* < 0.001, Greenhouse-Geisser corrected], indicating that the participants were more likely to perceive the auditory tokens closer to the /aba/ end of the continuum (BD1-BD3) as /aba/ and the tokens closer to the /ada/ end (BD7-BD9) as /ada/. Furthermore, no difference in the overall proportion of /aba/ responses was observed between the children with dyslexia (*M* = 0.51, *SD* = 0.06) and typically reading children (*M* = 0.54, *SD* = 0.11); [*t*(38) = -0.92, *p* = 0.36], indicating that the slope was equivalent in both groups. Figure 3B shows the same slopes for children with dyslexia and all of the control participants tested (*n* = 56), once again showing similar categorical perception in typically reading children and children with dyslexia. The goodness of fit estimation of the slopes reflected in partial R^2^ values was 0.99 in the dyslexic, matched as well as the entire control group.

### Recalibration and Adaptation Tasks

#### Matched Groups

**FIGURE 4:**
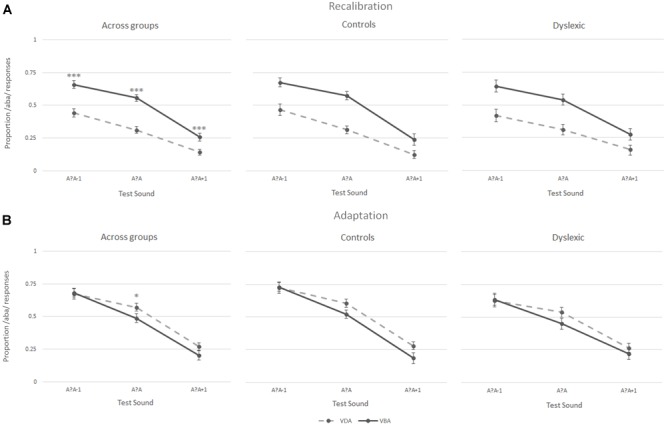
Results of the recalibration **(A)** and adaptation **(B)** tasks across groups (left), in typically reading children (middle) and in dyslexic readers (right); ^∗∗∗^*p* ≤ 0.001, ^∗^*p* ≤ 0.05.

During the recalibration task, participants’ perception of the three post-test sounds – the most ambiguous sound (a?a) and its two closest neighbors (a?a+1 and a?a-1) – was influenced by the preceding exposure blocks, as seen when analyzing the proportion of /aba/ versus /ada/ responses during the post-test trials. Intriguingly, both the children with dyslexia and the typically reading children showed a recalibration effect (Figure 4A middle and right columns, respectively). Thus, both groups were more likely to perceive the ambiguous post-test sounds as /aba/ following ‘aba’ exposure blocks (solid line Figure 4A). Similarly, ‘ada’ text shifted later perception toward /ada/ (dashed line Figure 4A). This effect was particularly pronounced for the most ambiguous /a?a/ sound (proportion of /aba/ responses children with dyslexia 0.54 vs. 0.31, typical readers 0.57 vs. 0.31, respectively). Across both groups, the participants only seemed to show a small adaptation effect for the most ambiguous post-test sound, namely the exposure to the clear /aba/ sound in combination with ‘aba’ text shifted the perception of the post-test trials to /ada/ (dashed line Figure 4B left). Correspondingly, being exposed to clear /ada/ in combination with ‘ada’ text led to a small shift in the perception of subsequent post-test trials toward /aba/ (solid line Figure 4B left).

A 2 (Task) × 2 (Exposure) × 3 (post-test sounds) × 2 (group) repeated measures ANOVA showed a significant task × exposure × post-test sounds interaction [*F*(2,76) = 3.52, *p* < 0.05] confirming that the participants responded differently to the post-test sounds following the two types of exposure blocks in recalibration and adaptation tasks. This was further confirmed by the significant main effects of task [*F*(1,38) = 31.27, *p* < 0.001], exposure [‘aba’ versus ‘ada’; *F*(1,38) = 8.65, *p* = 0.006], and post-test sounds [*F*(1,48) = 117.05, *p* < 0.001, Greenhouse-Geisser corrected], as well as significant task × exposure [*F*(1,38) = 45.32, *p* < 0.001], task × post-test sounds [*F*(1,61) = 3.38, *p* < 0.05, Greenhouse-Geisser corrected] and exposure × post-test sounds [*F*(2,76) = 7.39, *p* < 0.005] interactions. No main effect of group was observed [*F*(1,38) = 1.06, *p* = 0.31], and none of the interactions with group were significant (all *F* ≤ 2.1), corroborating the absence of significant differences in recalibration and adaptation results in children with dyslexia and typically reading children.

The results of the Recalibration task were further tested in a 2 (Exposure) × 3 (post-test sounds) × 2 (Group) repeated measures ANOVA. A main effect of exposure [*F*(1,38) = 51.43, *p* < 0.001], post-test sounds [*F*(1,49) = 84.02, *p* < 0.001, Greenhouse-Geisser corrected], as well as a significant exposure × post-test sounds interaction [*F*(2,76) = 8.37, *p* = 0.001] again highlighted that the participants responded differently to the post-test sounds depending on the type of exposure block preceding them. Results yielded no main [*F*(1,38) = 0.054, *p* = 0.81] or interaction (all *F* ≤ 0.9) effects for group.

A 2 (Exposure) × 3 (post-test sounds) × 2 (Group) repeated measures ANOVA was also run on the results of the adaptation task confirming the absence of an overall adaptation effect across sounds in both groups [*F*(1,38) = 2.35, *p* = 0.13]. The results revealed a main-effect of post-test sounds [*F*(1,52) = 80.73, *p* < 0.001, Greenhouse-Geisser corrected] and a non-significant trend toward an exposure × post-test sounds interaction [*F*(2,76) = 2.90, *p* < 0.06]. No other main effects or interactions were significant (all *F* ≤ 1.2).

*Post hoc* paired-samples *t*-tests were run on the proportion of /aba/ responses for each of the three post-test sounds following both exposure blocks (‘aba’ versus ‘ada’) in both tasks across groups. In the recalibration task, the analyses yielded significant differences in the proportion of /aba/ responses following an ‘aba’ exposure block compared to an ‘ada’ exposure block across all post-test sounds (/a?a/: *M* = 0.55, *SD* = 0.16 vs. *M* = 0.31, *SD* = 0.15, *t*(39) = 6.99, *p* < 0.001; /a?a/+1: *M* = 0.26, *SD* = 0.19 vs. *M* = 0.14, *SD* = 0.14, *t*(39) = 3.98, *p* < 0.001; and /a?a/-1: *M* = 0.66, *SD* = 0.18 vs. *M* = 0.44, *SD* = 0.20, *t*(39) = 6.43, *p* < 0.001). In the adaptation task, only the proportion of /aba/ responses to the most ambiguous sound (/a?a/) was significantly different following ‘aba’ versus ‘ada’ exposure blocks [*M* = 0.48, *SD* = 0.21 vs. *M* = 0.57, *SD* = 0.19, *t*(39) = -2.06, *p* < 0.05].

To test for potential response-strategies, a paired samples *t*-test was run on the proportion of /aba/ responses across all three post-test sounds in the recalibration task compared to the adaptation task (van Linden and Vroomen, 2008). The results revealed a significant difference in the proportion of /aba/ responses in the recalibration task (*M* = 0.57, *SD* = 0.50) compared to the adaptation task [*M* = -0.14, *SD* = 0.62;*t*(1,39) = 6.81, *p* < 0.001], indicating that the children did not employ a clear response strategy thus confirming the reliability of the observed recalibration effect.

#### Entire Control Group

**FIGURE 5:**
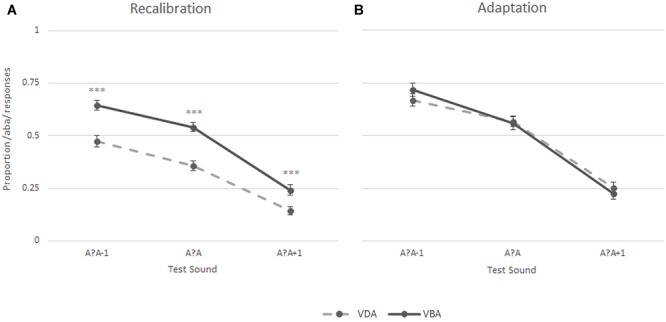
Results of the recalibration **(A)** and adaptation **(B)** tasks in the entire control group (*N* = 51); ^∗∗∗^*p ≤* 0.001.

The same analyses were also performed on the data of the entire control group and yielded similar recalibration results. Five of the 56 participants did not complete the adaptation task, thus the statistical analyses including the task condition are based on 51 participants. A 2 (Task) × 2 (Exposure) × 3 (post-test sounds) repeated measures ANOVA revealed a significant main effect of task [*F*(1,50) = 99.53, *p* < 0.001], exposure [‘aba’ versus ‘ada’; *F*(1,50) = 15.93, *p* < 0.001], and post-test sounds [*F*(1,69) = 155.55, *p* < 0.001, Greenhouse-Geisser corrected], as well as significant task × exposure [*F*(1,50) = 22.70, *p* < 0.001] and task × post-test sounds [*F*(1,67) = 0.82, *p* < 0.05, Greenhouse-Geisser corrected] interactions. The results are summarized in Figure 5, which illustrates that the participants showed a recalibration effect (solid line above the dashed line) but did not show an adaptation effect (no separation between the lines). A 2 (Exposure) × 3 (post-test sounds) repeated measures ANOVA was run for each task and revealed a significant main effect of exposure [*F*(1,50) = 41.09, *p* < 0.001], post-test sounds [*F*(1,70) = 128.02, *p* < 0.001, Greenhouse-Geisser corrected], and a significant exposure × post-test sounds interaction [*F*(2,100) = 4.45, *p* < 0.05] in the recalibration task as well as a main effect of post-test sounds [*F*(1,68) = 97.39, *p* < 0.001, Greenhouse-Geisser corrected] in the adaptation task, highlighting the presence of a recalibration effect and the absence of an adaptation effect.

*Post hoc* paired samples *t*-tests on the proportion of /aba/ responses for each of the post-test sounds per exposure block (‘aba’ versus ‘ada’) revealed significant differences in the proportion of /aba/ responses for each of the sounds following ‘aba’ compared to ‘ada’ recalibration exposure blocks [/a?a/: *M* = 0.52, *SD* = 0.15 vs. *M* = 0.33, *SD* = 0.16, *t*(50) = 5.80, *p* < 0.001; /a?a-1/: *M* = 0.62, *SD* = 0.17 vs. *M* = 0.46, SD = 0.19, *t*(50) = 5.30, *p* < 0.001; /a?a+1/: *M* = 0.24, *SD* = 0.17 vs. *M* = 0.14, *SD* = 0.13, *t*(50) = 4.34, *p* < 0.001]. None of the paired samples *t*-tests for the adaptation task yielded a significant result.

#### Relation With Standardized Reading Measures

Given the absence of overall group differences in recalibration, an important aspect to consider is whether the presence of this effect is related to individual differences in reading fluency, the magnitude of adaptation and/or the phoneme categorization slope. Accordingly, two separate linear regression analyses were performed in the matched groups, one to investigate potential links between the magnitude of the recalibration and adaptation effects (quantified as the proportion of /aba/ vs. /ada/ responses), the individual phoneme categorization slopes and standardized reading measures. The second analysis investigated the relation between the individual phoneme categorization slopes and the magnitude of the recalibration and adaptation effects and reading measures. Prior to running the regression analyses, the data were assessed for outliers using boxplots. In the matched groups, the analyses identified two outliers in categorization slope values, one child with dyslexia (lower quartile plus 3 times inter-quartile range) and one typically reading participant (lower quartile plus 1.5 times inter-quartile range). Similarly, 7 participants were identified as outliers in the entire control group according to the same criteria and were excluded from the subsequent regression analyses. All linear regression models initially included main effects for: group (dyslexia yes/no), recalibration and adaptation aftereffects, reading fluency and accuracy scores, and pre-test phoneme categorization slope values, as well as interactions between the main effects and dyslexia. Where applicable, these models were refined by removing interaction terms with a *p*-value exceeding 0.7 thus improving model fit. The reading measures were centered with respect to the overall average to facilitate interpretation.

**FIGURE 6:**
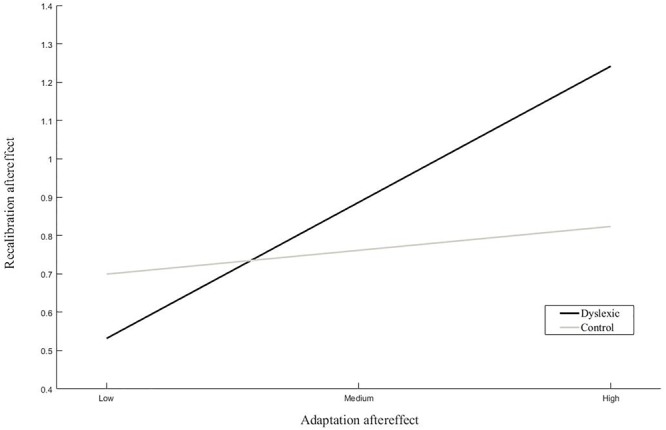
Simple slope analyses of the association between recalibration and adaptation. The average magnitude of the recalibration effect is plotted with respect to the relative magnitude of the adaptation aftereffect for low, average and high levels of adaptation within each group.

The results of the linear regression analyses of the magnitude of the recalibration effect showed a significant interaction between dyslexia and the adaptation effect (Table 2 ‘Recalibration effect’). Simple slope analyses of the interaction effect revealed a significant positive association between the strength of the recalibration and adaptation effects in children with dyslexia but not typically reading children (Figure 6). Moreover, a trend was observed in the main effect of pre-test slope on recalibration across groups. Regression analyses of the phoneme categorization slope values did not reveal significant main or interaction effects in the matched groups. However, the main effect of recalibration did approach significance, suggesting a link between pre-test slope and the strength of the recalibration effect (Table 2 ‘Pre-test slope’). Slope values were not found to significantly differ between children with dyslexia (*n* = 19) and typically reading children (*n* = 19; *t*(36) = -0.54, *p* = 0.59, equal variances assumed).

**Table 2:** Results of the recalibration effect and pre-test slope regression analyses in the matched groups.

Predictor	Beta estimate	*SE*	*t*-value	*p*
**Recalibration effect**				
Intercept	0.76	1.07	0.71	0.4827
Dyslexic	0.13	1.22	0.10	0.9189
Adaptation aftereffect	–0.10	0.20	–0.49	0.6264
Total Reading Fluency [T]	0.01	0.01	1.18	0.2490
Total Reading Accuracy [T]	–0.03	0.03	–1.00	0.3255
Slope	–**1.25**	**0.69**	–**1.82**	**0.0801**
Dyslexic^∗^Adaptation aftereffect	**0.66**	**0.27**	**2.43**	**0.0216**^∗^
Dyslexic^∗^Total Reading Fluency	–0.04	0.02	–1.57	0.1287
Dyslexic^∗^Total Reading Accuracy	0.04	0.03	1.33	0.1937
Dyslexic^∗^Slope	0.80	0.83	0.97	0.3424
**Pre-test slope**				
Intercept	0.15	0.27	0.56	0.5811
Dyslexic	0.22	0.55	0.41	0.6868
Adaptation aftereffect	0.12	0.09	1.32	0.1984
Recalibration aftereffect	–**0.15**	**0.08**	–**1.82**	**0.0792**
Total Reading Fluency [T]	–0.02	0.01	–1.65	0.1093
Total Reading Accuracy [T]	0.00	0.01	0.70	0.4871
Dyslexic^∗^Adaptation aftereffect	–0.18	0.13	–1.40	0.1724
Dyslexic^∗^Total Reading Fluency	0.02	0.01	1.54	0.1338
Dyslexic^∗^Total Reading Accuracy	–0.02	0.01	–1.34	0.1918

*The bold values indicate statistically significant results. ^∗^*p* ≤ 0.05.*

The regression analyses of the strength of the recalibration effect and phoneme categorization were also performed in the whole control group (*N* = 51), revealing a significant link between the strength of the recalibration effect and categorical perception of phonemes (Table 3 ‘Recalibration effect’ and ‘Pre-test slope’). Moreover, a significant association between reading accuracy and steepness of the pre-test slope was observed, with reading fluency scores also approaching significance (Table 3 ‘Pre-test slope’). These findings complement and extend those of the matched groups highlighting the influence of phoneme perception on recalibration, with the additional finding of a significant link between phoneme categorization and reading accuracy in the control group.

**Table 3:** Results of the recalibration effect and pre-test slope regression analyses in the entire control group.

Predictor	Beta estimate	*SE*	*t*-value	*p*
**Recalibration effect**				
Intercept	0.65	0.52	1.26	0.2138
Adaptation aftereffect	–0.05	0.14	–0.37	0.7147
Total Reading Fluency [T]	0.01	0.01	1.16	0.2527
Total Reading Accuracy [T]	–0.02	0.01	–1.48	0.1468
Slope	–**0.82**	**0.26**	–**3.13**	**0.0033**^∗∗^
**Pre-test slope**				
Intercept	0.04	0.29	0.12	0.9017
Adaptation aftereffect	–0.11	0.07	–1.51	0.1388
Recalibration aftereffect	–**0.24**	**0.08**	–**3.13**	**0.0033**^∗∗^
Total Reading Fluency [T]	**0.01**	**0.00**	**1.81**	**0.0782**
Total Reading Accuracy [T]	–**0.01**	**0.01**	–**2.10**	**0.0425**^∗^

*The bold values indicate statistically significant results. ^∗∗^*p* ≤ 0.005, ^∗^*p* ≤ 0.05.*

## Discussion

In the present study, we investigated reading-induced audio-visual plasticity in 8–10 year old children with dyslexia and typically reading children by using written text to recalibrate children’s perception of ambiguous speech sounds. Contrary to reported findings in adults, our results revealed that both groups of children reliably show a recalibration effect. The magnitude of the effect was significantly related to the magnitude of the adaptation effect in children with dyslexia but not typically reading children. Phoneme categorization slopes in turn revealed comparable categorization of /aba/ and /ada/ sounds in both children groups. Furthermore, extending the analyses to a sample of typically reading children of various reading levels revealed an association between phoneme categorization slope and reading accuracy. These findings emphasize the importance of studying different age groups to investigate a potential developmental trend in short-term text-induced audio-visual learning, and to uncover possible differences in mechanisms responsible for letter-speech sound coupling, phoneme perception and reading fluency in dyslexic and typical readers.

Replicating our recent findings in typically reading adults (Bonte et al., 2017; Keetels et al., 2018), our current findings show that text stimuli can successfully be used to bias the perception of ambiguous speech in 8–10 year-old children. Recalibration is proposed to rely on short-term perceptual learning mechanisms that help resolve the discrepancy between context information (e.g., lip-read speech, text) and ambiguous sound (Samuel and Kraljic, 2009; Vroomen and Baart, 2012). Unlike lip-read speech which is rooted in biology (Kuhl and Meltzoff, 1982), letter-speech sound associations are by nature arbitrary and are learnt through explicit instruction (Keetels et al., 2016; Fraga González et al., 2017). Our results suggest that already during the first years of reading acquisition, at least at the behavioral level, these learned associations lead to significant perceptual shifts similar to those induced by lip-read information (van Linden and Vroomen, 2008). That is to say, simple ‘aba’ and ‘ada’ syllables lead to perceptual recalibration in 8–10 year old children in the relatively transparent Dutch orthography that is characterized by fairly consistent letter-speech sound mappings and a rather small grain size. In future studies it would be interesting to test whether similar syllables also yield significant text-based recalibration in less transparent orthographies and/or orthographies with larger grain sizes (see e.g., Paulesu et al., 2000; Brennan et al., 2012; Lallier and Carreiras, 2017).

The observation of significant recalibration in children with dyslexia is in line with a previous study indicating comparable context sensitivity during speech perception in 7–9 year old children with dyslexia and typically reading children at auditory, phonetic and phonological levels (Blomert et al., 2004). But how can this observation be reconciled with the absence of a significant effect in adults with dyslexia (Keetels et al., 2018)? One possible explanation for the discrepancy between findings in children and adults is that 8–10 year-old children presumably have a wider integration window for letter-speech sound coupling. EEG research investigating letter-speech sound integration in children within our age range indicates timing differences in the MMN window in response to letter-speech sound pairs. Namely, unlike in adults, in children the audio-visual MMN effect is not restricted to simultaneous presentation of letters and speech sounds (Froyen et al., 2008), but is also seen when letters are presented 200 ms prior to the speech sounds. Furthermore, the MMN response peaks at a later time point, a pattern that gradually shifts to earlier and shorter integration windows with increased reading experience (Froyen et al., 2009; Žarić et al., 2014). These changes have been proposed to reflect the automatization of letter-speech sound coupling (Froyen et al., 2008, 2009). A similar pattern, albeit with a reduced sensitivity to letter-speech sound congruency and delayed with respect to their age-matched peers, is also observed in children with dyslexia (Froyen et al., 2011; Žarić et al., 2014, 2015). A wider temporal integration window might be beneficial when resolving the conflict between the ambiguous sound and disambiguating text, and may reflect how text to speech sound audio-visual learning mechanisms are still developing during the first few years of reading instruction. Furthermore, developmental changes in the sensitivity to text may follow an ‘inverted U’ trajectory, where text is a more salient stimulus in the first few years of reading instruction and the salience decreases with increased reading expertise (Maurer et al., 2008; Price and Devlin, 2011; Žarić et al., 2014, 2015; Fraga González, 2015). Because the children in our study fall within the age range of ‘peak’ text sensitivity, further observations of the same children in a longitudinal comparison may reveal interesting developmental trends in the text-based recalibration effect.

Another possibility that could explain the difference in results between the adults and children with dyslexia is that there might be larger inter-individual differences in adults. Thus, the adult dyslexic readers who do not show a text-based recalibration effect may suffer from a more severe form of dyslexia and/or may have switched to relying on different reading strategies circumventing one-to-one mappings of letters and speech sounds. Instead, reading is a daily occurrence for school-age children, with a presumably predominant reliance on letter-sound decoding skills especially for children with dyslexia included in our study who were at the initial phase of a dyslexia intervention with a focus on these skills.

Our results also contrast with previous findings reporting reduced sensitivity to letter-speech sound (in)congruency in children and adults with dyslexia (Blau et al., 2009, 2010; Froyen et al., 2009, 2011; Žarić et al., 2014, 2015; Karipidis et al., 2017). A possible reason for the observed differences in results may lie in the paradigms employed. While the aforementioned studies have used congruency manipulations and oddball paradigms to explore group differences between typical and dyslexic readers, we have used a more implicit measure. Recalibration typically involves the disambiguation of ambiguous speech signals based on short-term perceptual (audiovisual) learning. It is possible that, at a purely behavioral level, the task is not sensitive enough to capture subtle group differences between children with dyslexia and typically reading children. Indeed, previous studies on audiovisual integration have revealed underlying differences in brain mechanisms using neuroimaging methods despite a lack of significant differences in behavioral measures (see Nash et al., 2016; Plewko et al., 2018). In future studies it would be important to further understand the specific role of task and stimulus characteristics, as well as risk factors such as family history of dyslexia (Raschle et al., 2012; Plewko et al., 2018) in yielding these audio-visual integration deficits. Moreover a next essential step would be to combine our text-based recalibration paradigm with measurements of brain activity (e.g., Bonte et al., 2017) and investigate whether different or comparable neural mechanisms underlie the perceptual shifts in children with dyslexia and typically reading children.

Linear regression analyses of the magnitude of the recalibration effect revealed a significant association between recalibration and adaptation in the dyslexic but not typical readers. That is, in dyslexic readers, stronger recalibration was associated with stronger adaptation effects. Furthermore, in the matched groups, the recalibration effect showed a tendency toward an association with pre-test slope across participants. This link reached statistical significance when the analyses were extended to the entire control group, suggesting a close link between the categorical perception of phonemes and short-term text-induced audiovisual learning, with sharper phoneme categorization linked to stronger recalibration effects. The findings of the matched groups were thus extended and complemented by those of the entire sample of controls. We would therefore speculate that the abovementioned pattern of results would also replicate in a larger sample of both children with dyslexia and typically reading children.

Our study did not find support for proposed differences in categorical perception of speech sounds between children with dyslexia and typically reading children. This finding is in line with previous research reporting a similar lack of group differences (Blomert and Mitterer, 2004; Snellings et al., 2010) or differences only in small sub-groups of dyslexic readers (Manis et al., 1997; Joanisse et al., 2000), but not with others that do report reduced categorical perception of phonemes in dyslexic readers (Boets et al., 2011; Baart et al., 2012). While no significant association between phoneme categorization and reading measures was observed in the matched groups, the association between phoneme categorization and recalibration did approach significance. This relationship was confirmed by the results within the whole control group, revealing a significant link between the magnitude of the recalibration effect and the individual phoneme categorization slopes. Additionally, a significant link between reading accuracy and phoneme categorization also emerged in the entire control group, corroborating previous findings indicating that speech perception and reading are mediated by children’s phonological skills (Mcbride-chang, 1996) and that speech perception and phonological awareness measures are significant predictors of first grade reading accuracy in preschoolers (Boets et al., 2008). These findings warrant further investigation in a larger sample of dyslexic and typical readers.

Our data also revealed a small adaptation effect, with the /aba/ response proportions to the most ambiguous sound in the adaptation task reaching statistical significance across the dyslexic and typical readers. The main purpose of this task was to investigate potential response strategies and ensure the reliability of the observed recalibration effect (van Linden and Vroomen, 2008). The finding that children showed a shift in the perceptual boundary of the ambiguous post-test sounds in the direction of text in the recalibration but not adaptation task reaffirms the robustness of the recalibration effect across groups. Thus, if children had simply responded in line with the text seen during the exposure blocks for both tasks, there would be no significant difference in the proportion of /aba/ responses between recalibration and adaptation. The finding that the adaptation effect itself was only significant when both groups of children were pooled together and only for the most ambiguous sound likely reflects the previously observed developmental trend in adaptation (Sussman and Carney, 1989; Sussman, 1993; van Linden and Vroomen, 2008). Another potential explanation for the lack of adaptation effects in our study may be found in the proposed more fragile nature of the effect. While recalibration effects can already be observed after single exposure (Keetels et al., 2016), adaptation effects have been shown to develop after a longer time period, require more exposure trials to emerge, and be longer-lasting compared to recalibration effects (Vroomen et al., 2004, 2007).

## Conclusion

The present study investigated text-induced changes in perception of ambiguous speech sounds in children employing text-based recalibration. Our results indicate that both 8–10 year-old dyslexic and typical readers show significant text-induced shifts in their perception of ambiguous speech. This finding is likely rooted in the flexibility of the cortical systems for letter-speech sound integration which have not yet been ‘set in stone’ at this age and are thus more flexible in terms of phonemic category perception. Furthermore, the magnitude of the recalibration effect was linked to the adaptation effect in children with dyslexia but not in typical readers. Extending these analyses to a larger sample of only typical readers revealed additional associations between recalibration and phoneme categorization as well as phoneme categorization and reading measures. Our findings highlight the importance of considering task demands and dynamic developmental changes in reading, speech perception and audiovisual learning when investigating group differences between typical and dyslexic readers. Future longitudinal research following the same children at different stages using both behavioral and brain activity measures is thus essential to understand the neurocognitive mechanisms explaining individual differences in acquired reading levels and dyslexia.

## Author Contributions

MB and LR designed the experiments. LR and RJ collected and analyzed the data. MB, LR, and RJ wrote the paper.

## Conflict of Interest Statement

The authors declare that the research was conducted in the absence of any commercial or financial relationships that could be construed as a potential conflict of interest.

## Footnotes

1 https://www.reallusion.com/
